# Understanding Hip Contact Stress Based on Types of Physical Activity: A Systematic Review

**DOI:** 10.1002/hsr2.70305

**Published:** 2025-01-22

**Authors:** Arief Indra Perdana Prasetya, Muhammad Imam Ammarullah, Tri Indah Winarni, Adriyan Pramono, Jamari Jamari, Tunku Kamarul, Ardiyansyah Syahrom

**Affiliations:** ^1^ Department of Surgery Sub Orthopaedic and Traumatology Faculty of Medicine Universitas Islam Sultan Agung Semarang Central Java Indonesia; ^2^ Postgraduate Program, Faculty of Medicine Universitas Diponegoro Semarang Central Java Indonesia; ^3^ Department of Mechanical Engineering Faculty of Engineering Universitas Diponegoro Semarang Central Java Indonesia; ^4^ Undip Biomechanics Engineering & Research Centre (UBM‐ERC) Universitas Diponegoro Semarang Central Java Indonesia; ^5^ Department of Anatomy Faculty of Medicine Universitas Diponegoro Semarang Central Java Indonesia; ^6^ Center for Biomedical Research (CEBIOR), Faculty of Medicine Universitas Diponegoro Semarang Central Java Indonesia; ^7^ Department of Nutrition Science Faculty of Medicine Universitas Diponegoro Semarang Central Java Indonesia; ^8^ Department of Orthopaedic Surgery (NOCERAL) Faculty of Medicine Universiti Malaya Kuala Lumpur Federal Territory of Kuala Lumpur Malaysia; ^9^ Department of Applied Mechanics and Design School of Mechanical Engineering Universiti Teknologi Malaysia Skudai Johor Malaysia; ^10^ Mechanical Engineering and Medical Device and Technology Centre (MEDITEC) Universiti Teknologi Malaysia Skudai Johor Malaysia

**Keywords:** hip contact stress, hip osteoarthritis, physical activity, walking speed

## Abstract

**Background and Aims:**

High contact stresses involving the hip have been shown to increase the risk of developing hip osteoarthritis (OA). Although several risk factors have been identified for OA, a holistic approach to predicting contributed factors toward increased hip contact stresses have not been explored. This study was conducted to comprehensively understand the effects of physical activity on high hip contact stress as predisposing factors of OA.

**Methods:**

The protocol of this systematic review was registered in PROSPERO with registration number CRD42022296638 and conducted based on PRISMA guidelines. Full articles that matched our inclusion criteria were selected using PubMed, Web of Science, and Scopus search engines and keywords such as “hip contact stress,” “hip contact force,” and/or “hip contact pressure.” Category of factors, experimental design, results of the study, and evidence from each article were analyzed.

**Results:**

In total 7972 papers were screened, identified, and reviewed. Two independent authors read the collected fulltext of eligible articles resulting in 21 papers that fulfilled the inclusion criteria of this systematic review.

**Conclusion:**

Types of physical activity (*n* = 21) have correlation with high hip joint contact stress in various manner. Based on the research findings obtained from various inclusion papers, it can be broadly concluded that the more intense the physical activity, such as running and stair climbing, the greater the impact on the increase in hip contact stress values. However, the reviewed studies vary in their methods. This finding suggested that this area is not well investigated and warrants future research.

**Abbreviations**
OA: osteoarthritis
PRISMA: Preferred Reporting Items for Systematic Reviews and Meta‐Analyses

## 1 Background

Osteoarthritis (OA) is an intriguing joint condition that warrants study due to its impact on functional ability and quality of life [1, 2]. Dieppe explains that OA involves progressive joint damage resulting from degenerative processes and biomechanical factors acting on the joint [3]. Biomechanical alterations have remained a subject of interest for researchers seeking to gain a deeper understanding of the various factors that can influence joint biomechanics, ultimately leading to a heightened risk of OA [2, 3].

The hip joint, with its ball‐and‐socket shape, possesses the remarkable capacity for multidirectional movement [4, 5]. Additionally, this weight‐bearing joint frequently falls prey to the development of OA owing to these distinctive characteristics [2, 4]. Hip joint contact stress is an extensively studied aspect of biomechanics. Numerous studies and reports have explored the predictive modeling of hip contact stress at different points [6, 7]. Physical activity involved hip joint has direct influence to the magnitude of hip contact stress. Numerous studies have been conducted to quantify hip contact stress in various body positions and during daily activities involving body weight or predetermined loads [8, 9, 10, 11]. Due to the variation in methods and results from previous studies concerning the impact of different activities on the magnitude of hip contact stress, this systematic review aims to examine the association between the type of physical activity and hip contact stress, as well as the relationship between walking or running speed and the resulting contact stress.

## 2 Methods

A systematic review was conducted following the guidelines outlined by Preferred Reporting Items for Systematic Reviews and Meta‐Analyses (PRISMA). Before the commencement of the official search, the proposed keywords were cross‐referenced with the PROSPERO database to ascertain whether a similar systematic review had previously been published or registered. No pertinent results were discovered in this search. The literature search was carried out in accordance with the PRISMA guidelines and extended until December 31, 2023. The study protocol was registered with the International Prospective Register of Systematic Reviews (PROSPERO) under the registration number CRD42022296638.

After a thorough preliminary assessment of the literature keywords, title, and abstract from three databases (Web of Science, SCOPUS, and PubMed) related to “hip contact stress” OR “hip contact force” OR “hip contact pressure.” The retrieved references were subsequently subject to additional keyword, title, and abstract searches to collect the eligible articles. Further searching was carried out using the word “physical activity” OR “daily activity.”

Inclusion criteria were as follows: (i) complete and original studies in humans; (ii) quantitative analyses of various physical activities (sitting, walking, standing, and ascend/descend the stair); (iii) the factors mentioned in “(ii)” should be related to hip contact stress calculation. Exclusion criteria were as follows: (i) nonhuman study, (ii) non‐hip joint study, (iii) implant study, (iv) post‐arthroplasty sample, and (v) pathological conditions other than primary hip OA (e.g., post‐traumatic, post‐sequalae of infection, related to congenital/anatomical anomaly, and tumor).

The articles were selected by two independent reviewers. Initially, duplicates, editorials, case studies, incomplete articles, and non‐original investigations were excluded. After screening, the titles that did not satisfy our selection criteria were excluded. After all the selected abstracts were read, the articles that could potentially fit to the inclusion/exclusion criteria were evaluated in their full format. A final independent decision was made by the reviewers. We did not conduct a bias test on the selection results of the eligible journals. Any differences observed after the final results were discussed and consulted with the supervisor and involved experts. The list of references cited in each article was screened for potential inclusion in this review.

Graphs or diagrams containing related data were obtained using an online graphic extractor (Automeris plot interpreter) and also were used to recalculate and predict the statistical data from these graphs or histograms. Statistical methodology in this study involved synthesizing data extracted from the included studies. Hip contact stress values were collected directly from the reported data in each study or calculated based on formulas and values provided in the original articles. The results were then compiled and analyzed descriptively to identify patterns and trends in hip contact stress associated with various physical activities. Variability in reported methods across studies limited the use of more advanced statistical analyses, such as meta‐analysis, and the findings were interpreted qualitatively. After the statistical values were collected, further calculation was performed to generate uniform values of hip contact stress for the analysis of the combined effect among these 21 eligible studies. After the values of force acting in the hip joint were collected from each paper, further calculation based on data obtained from those papers was performed to generate uniform values of hip contact stress for the analysis of the pattern of physical activity impact on hip contact stress.

Confidence intervals for effect estimates and *p* values were not provided, as this study did not involve direct statistical analysis or hypothesis testing. Instead, the findings were synthesized descriptively, highlighting patterns and trends observed across the included studies.

## 3 Results

**Figure 1:**
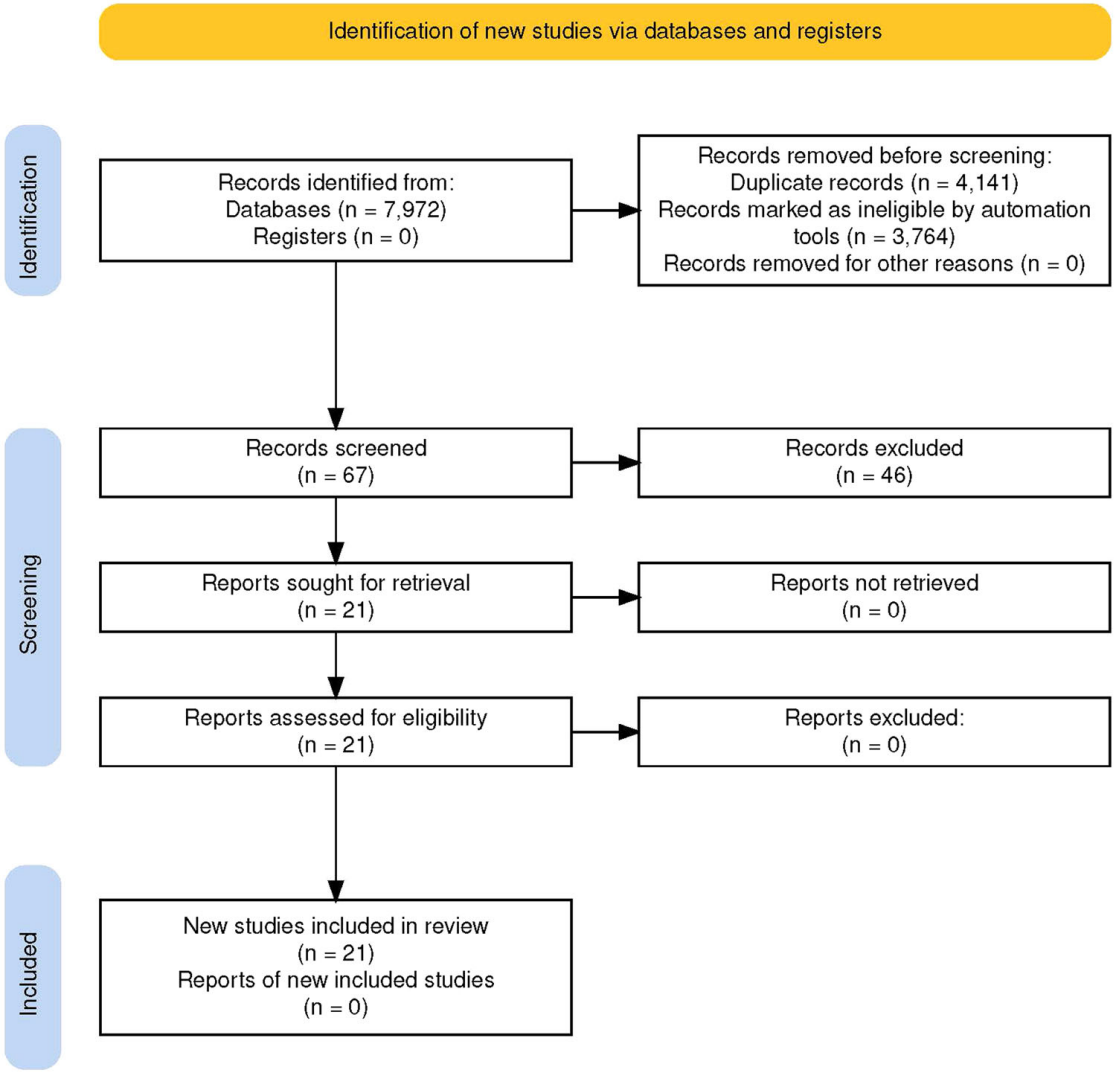
Systematic review flow chart diagram.

Up to the submission date of this review, no systematic review addressing the relationship of hip joint parameter and physical activity with hip joint contact stress has been registered or published. The first identification stage was to search papers containing “hip contact stress” OR “hip contact force” OR “hip contact pressure” as the keyword from PubMed, Scopus, and Web of Science databases then export them into EndNote 20 reference manager. We collected total of 7972 papers from those three databases which are consisting of 779 papers containing “hip contact force” from PubMed, 436 papers containing “hip contact pressure” from PubMed, 733 papers containing “hip contact stress” from PubMed, 1415 papers containing “hip contact force” from SCOPUS, 746 papers containing “hip contact pressure” from SCOPUS, 1208 papers containing “hip contact stress” from SCOPUS, 1261 papers containing “hip contact force” from Web of Science, 654 papers containing “hip contact pressure” from Web of Science, and 740 papers containing “hip contact stress” from Web of Science. We found 6271 duplicates (double or triple copies from each journal from those three databases). After removing the duplicates, we collected 3831 remaining papers. Only 67 papers remains after further search using “daily activity” OR “physical activity.” These papers then grouped as “Physical Activity on Hip Contact Stress.” The remaining papers were assessed using the inclusion and exclusion criteria. And resulting 21 papers fulfilled those criteria (Figure 1).

Table 1 encompasses 21 papers that highlight the impact of daily physical activities on hip joint contact stress, based on the studies included in the analysis.

**Table 1:** Summary of included studies assessing physical activity to hip contact stress.

No.	Author (year)	Title	Sample/event size	Procedure	Aspect of activity	Aspect of hip contact stress
1	van den Bogert et al. (1999) [12]	An analysis of hip joint loading during walking, running, and skiing	9 male models	Analyzing hip contact force during walking (1.5 m/s), running (3.5 m/s), and various type of skiing using accelerometer and based on hip joint moment and lever arm	Walking, running, skiing (Alpine skiing in several styles)	Hip contact force
2	Assassi and Magnenat‐Thalmann (2016) [13]	Assessment of cartilage contact pressure and loading in the hip joint during split posture	11 ballet dancer models	Finite element analyses of hip contact pressure (Mpa), hip contact area (%), and hip contact force (%BW), during walking, standing, and split position	Walking, standing, splitting	Hip contact force, hip mean contact stress
3	Sangeux (2019) [5]	Biomechanics of the hip during gait	1 computerized model in 8 activities simulation	Analyzing and simulating hip biomechanics and hip contact force (based on Bergmann et al.)	Cycling, sitting down, standing up, squatting, walking, walking upstairs, walking downstairs, jogging	Hip contact force
4	Heller et al. (2005) [14]	Determination of muscle loading at the hip joint for use in preclinical testing	1 computerized model simulating walking and walking upstairs	Analyzing hip contact force during walking and stair climbing using computerized model	Walking, walking upstairs (in several % of stance phase of the gait cycle)	Hip contact force
5	Anderson et al. (2010) [15]	Effects of idealized joint geometry on finite element predictions of cartilage contact stresses in the hip	6 finite element models	Analyzing hip joint contact stress during several activities on subject‐specific FE models compared with a simple‐modified model	Walking, walking upstairs, and walking downstairs	Hip mean contact stress, hip peak contact stress
6	Altai et al. (2021) [16]	Femoral neck strain prediction during level walking using a combined musculoskeletal and finite element model approach	5 models	Analyzing femoral neck strain during walking using combination of FEM and computerized mechanical model	Walking (in several % of the gait cycle)	Hip contact force
7	Deng et al. (2018) [17]	Femoral neck stress in older adults during stair ascent and descent	17 models (7 males, 10 females)	Comparing hip contact force during stair ascent and stair descent using computerized hip model 17 models based on 7 males and 10 females	Walking upstairs and walking downstairs (in several % of stance phase of the gait cycle)	Hip contact force, hip peak contact stress
8	Harris et al. (2012) [18]	Finite element prediction of cartilage contact stresses in normal human hips	10 models	Analyzing cartilage contact stress of hip joint using FE based on 10 sample	Walking (several phase of gait), walking upstairs, walking downstairs	Hip contact force, hip mean contact stress, hip peak contact stress
9	Henak et al. (2013) [19]	Finite element predictions of cartilage contact mechanics in hips with retroverted acetabula	20 models (10 normal acetabulum, 10 retroverted acetabulum)	Analyzing hip contact stress of hip joint with normal acetabulum in comparation to retroverted acetabulum using finite elements models	Walking, walking upstairs, walking downstairs, chair rise	Hip peak contact stress, hip mean contact stress, contact area
10	Daniel et al. (2008) [20]	hip contact stress during normal and staircase walking: the influence of acetabular anteversion angle and lateral coverage of the acetabulum	1 mathematical models with modification of femoral anteversion and center edge angle	Analyzing hip center edge angle and femoral anteversion to determine hip contact pressure during several activities	Walking (in several % of gait phase), walking upstairs, walking downstairs	Normalized peak contact stress on body weight
11	Heller et al. (2001) [21]	influence of femoral anteversion on proximal femoral loading: measurement and simulation in four patients	Computerized model from four models	Analyzing proximal femoral loading based on femoral anteversion variation during several activities	Walking (% of gait phase), walking upstairs	Hip contact force
12	Duda, Schneider, and Chao (1997) [22]	Internal forces and moments femur during walking	1 model to analyze in several methods during activity	Describe the internal load state acting at different levels along the human femur during various phases of gait	Walking (% of gait phase)	Hip contact force
13.	Schache et al. (2018) [23]	Is running better than walking for reducing hip joint load	8 models adapted from eight participants (4 male, 4 females)	Analyzing and comparing hip contact force during walking and running	Walking (various steady state), running	Ground reaction force, hip joint contact force
14.	Costigan, Deluzio, and Wyss (2002) [24]	Knee and hip kinetics during normal stair climbing	35 students (15 males, 20 females) without any hip and knee complaints	Analyzing reaction force of the hip and knee joint using markers to measure estimated muscle force	Walking, walking upstairs	Hip reaction force, hip joint moments
15	Giarmatzis et al. (2015) [25]	Loading of hip measured by hip contact force at different speeds of walking and running	20 participants (10 males, 10 females)	Analyzing hip contact force using markers (50 retro‐reflective markers) to measure kinematic force and construct musculoskeletal modeling	Walking (various speeds)	Hip contact force, ground reaction force, hip joint moment
16	Debevec et al. (2010) [26]	One‐legged stance as a representative static body position for calculation of hip contact stress distribution in clinical studies	1 geometrical model representing normal walking (based on Bergmann study)	Analyzing hip joint reaction force during one‐leg stance and walking	One‐leg stance, walking	Normalized hip contact stress on body weight
17	Pellikaan et al. (2018) [27]	Ranking of osteogenic potential of physical exercises in post‐menopausal women based on femoral neck strains	14 musculoskeletal modeling and Finite Elements Modeling adapted from 14 post‐menopausal elderly women	Analyzing hip contact force (proximal femur) on osteoporotic condition in relation to post‐menopausal condition	Walking (various speeds), running, hopping, resistance training exercise	Normalized hip contact force on body weight (additional measurements: femoral displacements, peak tensile strains, peak compressive strains)
18	Henak et al. (2013) [28]	Specimen‐specific predictions of contact stress under physiological loading in the human hip: validation and sensitivity studies	5 finite elements models based on 5 cadaveric male models	Analyzing hip contact stress on several hip joint anatomical regions during various loading in the representation of walking downstairs	Walking downstairs (with various loading)	Hip peak contact stress, hip mean contact stress
19	Martell et al. (2014) [29]	Strain energy in the femoral neck during exercise	1 finite elements model generated from 1 cadaveric female on 15 weight‐bearing activities	Analyzing energy working on femoral neck during several activities	Chair up/down, step up, squat, squat with weight, walking upstairs, walking downstairs, walking (1.3 m/s), long jump, vertical jump, hip isokinetic motion	Hip joint reaction force (additional measurements: normalized strain energy on body weight)
20	Wang et al. (2005) [30]	The hip stress level analysis for human routine activities	1 computerized model on 8 different activities	Analyzing hip contact stress during eight daily activities using computerized modeling	Slow walking, normal walking, fast walking, walking upstairs, walking downstairs, standing up, sitting down, knee bend	Hip peak contact stress
21	Xiong et al. (2022) [31]	Changes in hip joint contact stress during a gait cycle based on the individualized modeling method of “gait‑musculoskeletal system‑finite element”	2 computerized model based on 1 male and 1 female	Analyzing hip joint dynamics characteristics and the changes in the hip contact stress during gait cycle	Walking (% gait cycle)	Hip peak contact stress

### 3.1 Physical Activity in Correlation With Hip Contact Stress

**Figure 2:**
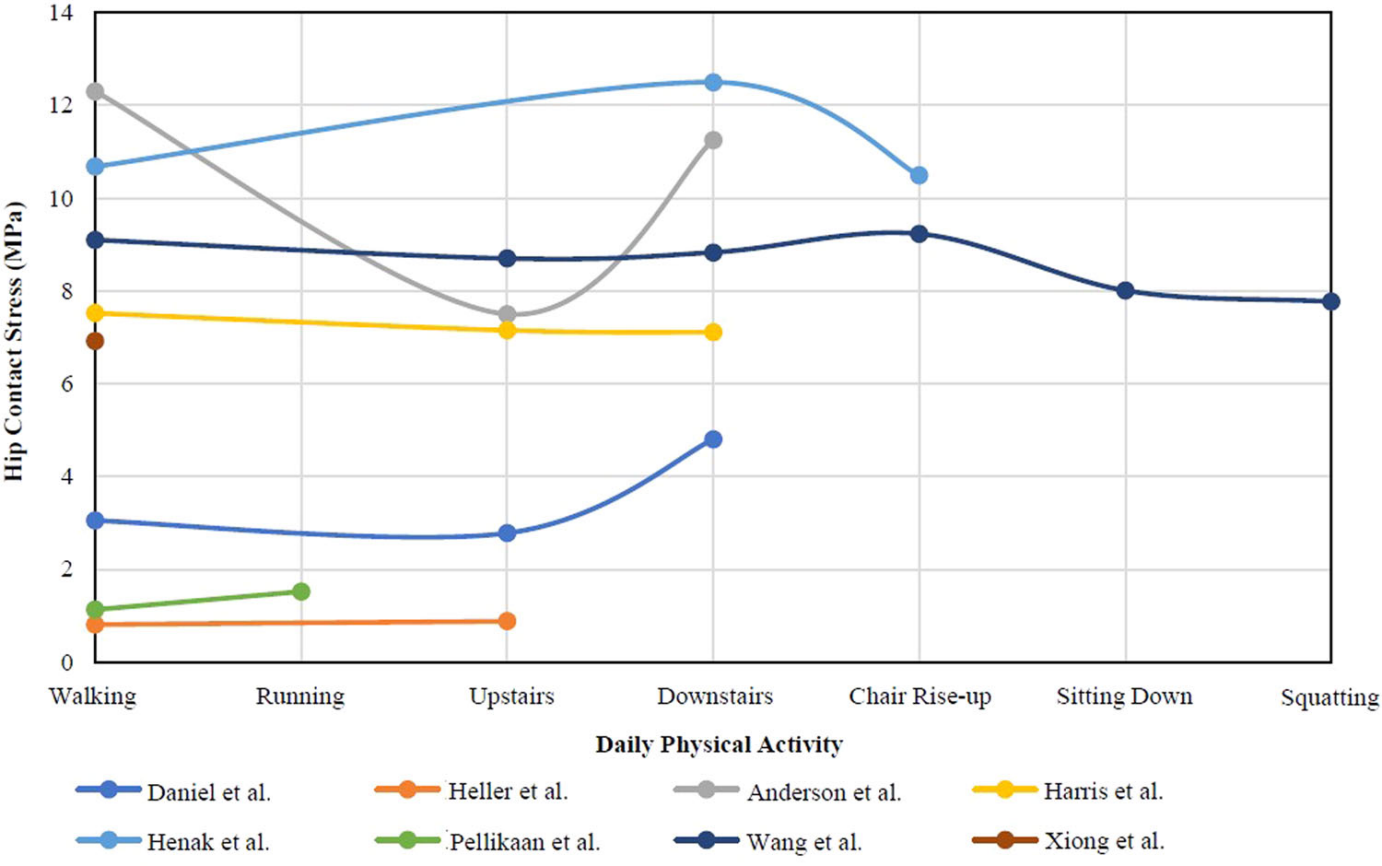
Daily physical activity on hip contact stress (MPa).

**Figure 3:**
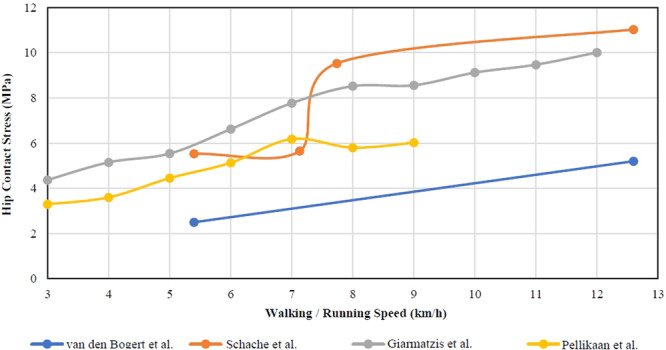
Walking/running speed (km/h) on hip contact force (BW).

Twenty‐one papers were collected in this group. Figure 2 shows eight studies analyzed the effect of several daily activities on hip contact stress in various result. Four studies analyzed the effect of walking/running speed on hip contact stress (Figure 3). The presence of bias in the results of hip contact stress calculations obtained from each inclusion paper might occur due to differences in methodology, sample variations, the units of force used, or the types of activities studied in each research. We performed further calculations using several formula to generate uniform values of hip contact stress.

## 4 Discussion

In this systematic review, we identified a number of physical activities that are closely linked to hip contact stress. Numerous studies have been given to understanding hip biomechanics, particularly, in the context of hip contact stress. The management of contact stress is contingent on the extent to which loads are distributed across the hip articular surface [32, 33, 34]. It is widely theorized that body weight, along with any additional loads, significantly contributes to hip joint contact stress [35, 36]. Engaging in activities that involve carrying heavy objects and/or assuming different joint positions can potentially introduce additional loads and alter the biomechanical aspects as well [30, 37]. Previous studies have sought to investigate the essential role played by body weight and physical activity in influencing hip joint contact stress [13, 30].

In a standing position, the hips bear the weight of the head, trunk, and upper limbs, which accounts for approximately 62% of an individual's body weight. The body's center of gravity is approximately aligned with the vertical axis passing through the heads of the femurs, slightly above the midpoint of the axis connecting these two points. In this position, the maintenance of balance requires minimal or negligible muscular effort. Assuming the lower limbs are of equal length, each hip joint carries approximately 31% of the body's weight. This force acts vertically and symmetrically on both sides of the hip joint [18, 38].

When standing on one leg, the hip on that side carries the weight of the head, trunk, upper leg, and lower leg on the other side. This weight is concentrated at the center of gravity and is exerted on the hip as an axial load. The axial load is typically calculated as 81% of the total body weight. The center of gravity of the whole body aligns vertically with the foot acting as a support on the ground. The center of gravity of the individual body parts is located further away from the loaded hip. The axial load causes eccentric forces in the hip, which can result in tilting of the hip toward adduction in relation to the femur [26, 30]. During walking, each hip supports a portion of the body weight, including the head, trunk, upper limbs, and legs that are swung during each period of single support. This repetitive force on the hip during the gait cycle can be likened to swinging a hammer or performing an oscillatory movement. This force oscillates near the coronal plane at the hip joint. Additionally, other complex biomechanical processes come into play during activities such as running, sitting, squatting, and walking up and downstairs, each with their own unique forces on the hip joint [5, 20, 31].

With regard to physical activity as contributing factors to determine value of hip joint contact stress, we performed this systematic review to analyze the results of previous studies.

### 4.1 Physical Activity on Hip Contact Stress

In this systematic review, 21 articles were selected to examine the role of physical activity on hip joint contact stress. These studies focused on analyzing the forces exerted on the hip joint during various activities such as walking, running, and ascending and descending stairs. The results revealed variations in hip contact force, mean hip contact stress, and peak hip contact stress (Figure 2). Some studies also investigated the forces at different phases of walking. Additionally, an analysis was conducted to determine the correlation between walking and running speed and hip CF. The findings indicated that higher speeds of walking or running result in increased hip CF, as depicted in Figure 3.

Walking speed can have an effect on hip contact stress, but the relationship is complex and influenced by various factors. Generally, walking at a faster speed increasing the force and load on the hip joint, potentially leading to higher contact stresses. This is because a faster walking pace leads to a higher impact force and greater muscle force on hip joint during step [39, 40]. However, it is important to note that the hip joint is a complex structure influenced by multiple factors, such as individual anatomy, joint health, and biomechanics. Other factors, like body weight, stride length, and gait mechanics, can also affect hip contact stress. Moreover, the body has adaptive mechanisms that can help mitigate the impact of higher walking speeds on hip joint loading [23, 25].

This systematic review has several limitations. First, the methodological heterogeneity among the included studies, ranging from variations in sample populations to differences in experimental designs and units of measurement poses challenges in synthesizing consistent conclusions. Second, data extraction from graphs and recalculations may have introduced minor errors, which could influence the derived values of hip contact stress. Third, the absence of a quantitative bias assessment further limits the robustness of our findings. Finally, the studies included lacked representation of diverse anthropometric parameters, such as body mass index, which could significantly impact hip contact stress values. These limitations emphasize the need for future research to adopt standardized methodologies and to investigate underexplored variables affecting hip biomechanics.

## 5 Conclusion

Types of physical activity contribute in determining hip joint contact stress, although, their constant and combined effects on hip contact stress were still not clearly determined due to different parameters of interest. Different physical activity leads to different hip joint contact stress. However, this systematic review has limitations due to various methods and units/types of force that act on the hip joint from each paper, we are unable to determine the combined and constant effect of each study.

## Author Contributions

**Arief Indra Perdana Prasetya:** data curation, formal analysis, investigation, software, writing–original draft. **Muhammad Imam Ammarullah:** project administration, software, validation, visualization, writing–review and editing. **Tri Indah Winarni:** conceptualization, funding acquisition, resources, supervision, writing–review and editing. **Adriyan Pramono:** funding acquisition, investigation, validation, visualization, writing–review and editing. **Jamari Jamari:** funding acquisition, resources, software, supervision, writing–review and editing. **Tunku Kamarul:** methodology, supervision, writing–review and editing. **Ardiyansyah Syahrom:** methodology, supervision, writing–review and editing.

## Disclosure

The authors declare that this manuscript is original, has not been published before and is not currently being considered for publication elsewhere. The authors confirm that the manuscript has been read and approved by all named authors and that there are no other persons who satisfied the criteria for authorship but are not listed. The authors further confirm that the order of authors listed in the manuscript has been approved by all of us. The authors understand that the corresponding author is the sole contact for the Editorial process. The corresponding author is responsible for communicating with the other authors about progress, submissions of revisions, and final approval of proofs.

## Ethics Statement

This study does not involve human participants or animals, and ethical approval was not required. All research procedures adhered to relevant ethical guidelines and best practices for nonhuman and nonanimal research.

## Consent

The authors consent to the publication of this manuscript.

## Conflicts of Interest

The authors declare no conflicts of interest.

## Transparency Statement

The authors affirm that this manuscript is an honest, accurate, and transparent account of the study being reported; that no important aspects of the study have been omitted; and that any discrepancies from the study as planned (and, if relevant, registered) have been explained.

## Data Availability

The necessary data used in the manuscript are already present in the manuscript.
